# Periodization in Orthobiologics Rehabilitation

**DOI:** 10.3390/jcm15052006

**Published:** 2026-03-05

**Authors:** Georgios Kakavas, George Skarpas, Trifon Totlis, Panagiotis Kouloumentas, Nikolaos Malliaropoulos, Florian Forelli

**Affiliations:** 1 Fysiotek Spine & Sports Lab, 17562 Athens, Greece; georgios.kakavas@gmail.com; 2 Orthopaedics, Hellenic Open University, 10677 Athens, Greece; 3 MIS Orthopedic Center, 54622 Thessaloniki, Greece; totlis@auth.gr; 4 Aristotelian University, 54622 Thessaloniki, Greece; 5 Athens Sport Orthopaedics, 10677 Athens, Greece; 6 Thessaloniki Sports Medicine Clinic, 54622 Thessaloniki, Greece; contact@sportsmed.gr; 7 Sports Clinic, Rheumatology Department, Barts Health NHS Trust, London E1 1BB, UK; 8 Haute-Ecole Arc Santé, HES-SO University of Applied Sciences and Arts Western Switzerland, 2800 Delémont, Switzerland; 9 Orthopedic and Surgery Department, Ramsay Healthcare, @OrthoLab, Clinic of Domont, 95330 Domont, France; 10 SFMKS-Lab, Société Française des Masseurs-Kinésithérapeutes du Sport, 93380 Pierrefitte sur Seine, France

**Keywords:** orthobiologics, periodization, neuroplasticity, rehabilitation, mesocycles

## Abstract

Orthobiologic treatments such as platelet-rich plasma and stem cell therapies are increasingly used to support the healing of tendons, ligaments, and joints. This perspective proposes applying periodization—a structured, progressive model borrowed from athletic training—to rehabilitation following orthobiologic interventions in order to improve functional outcomes. The framework is organized into sequential phases that align with biological stages of healing. Early phases emphasize pain control, inflammation management, and safe, controlled mobility. Rehabilitation then progresses toward gradually increasing load bearing and strength, and toward more specific exercises to promote tissue regeneration while reducing the risk of re-injury. In later phases (mesocycles), the model highlights the importance of neuroplastic adaptations for sustained functional recovery, including neurogenesis, synaptic plasticity, and functional remodeling to safer RTP for athletes. A key advantage of this approach is its adaptability: progression can be individualized according to a patient’s recovery trajectory and response to loading. By aligning rehabilitation progression with intrinsic healing processes and integrating physiological and neuromuscular goals, the proposed model aims to maximize regenerative potential across both athletic and non-athletic populations. Overall, this neuroplastic periodized approach offers a practical, evidence-informed structure to guide clinicians in delivering patient-centered regenerative rehabilitation and may help standardize care after orthobiologic procedures.

## 1. Introduction

Regenerative medicine, and its orthobiologics subsection, are widely debated, yet growing evidence indicates that certain regenerative medicine products are safe and efficacious in treating sports injuries [1]. A multidisciplinary team of orthopedic surgeons, sports medicine physicians, fitness professionals, and physical therapists must advise each patient on the best treatment approach.

Orthobiologics are used in acute injuries and chronic conditions affecting muscles, bones, joints, and soft tissues (ligaments, tendons, and cartilage) [2]. Physicians use autologous or heterologous cells and growth factors to improve recovery after an injury or minimize damage from degenerative conditions, such as osteoarthritis.

The most common cell and tissue types used in regenerative medicine are: Adipose (fat) tissue, bone marrow, and blood plasma.

Orthobiologic therapy uses platelets or cells from the patient’s own body to support healing after an injury. The term ‘blood-derived products’ refers to a wide range of products obtained by processing peripheral blood using various systems/techniques, yielding blood fractions enriched in therapeutic molecules. Among them, the best known and most commonly used are Platelet Rich Plasma (PRP), platelet-rich fibrin, platelet-rich growth factors, autologous conditioned plasma, and autologous protein solution, all based on platelet concentration, as well as other products such as autologous conditioned serum (ACS) and alpha-2-macroglobulin (A2M). Orthobiologics are directly injected into the injured joint, ligament, tendon, bone, or cartilage [3]. Once injected, these substances exert their anti-inflammatory and regenerative effects, helping minimize pain, promote healing, and improve joint function.

After the application of an orthobiologic device, physical therapists design programs that include several components, such as endurance, flexibility, proprioception/kinesthesia, balance, joint and soft-tissue mobility, speed, and power [4]. These plans often follow a logical sequence to promote optimal healing and restore peak performance. A significant challenge for sports physical therapists is designing optimal training programs that facilitate neural and muscular adaptations while accounting for biological constraints on healing and athlete safety [5].

This perspectives the provided periodization model and discusses the potential pros and cons of each rehabilitation approach. It then provides a sample program or structural framework for the various approaches described herein, for the sports physical therapist to implement in rehabilitation following orthobiologics applications. Finally, a review of programming to maximize the neural component of rehabilitation will be discussed, as this variable is most critical not only for the recovering athlete but also for healthy, trained, or untrained orthopedic patients.

In this Perspective, we operationally define regenerative rehabilitation as the intentional integration of an orthobiologic intervention with rehabilitation strategies (education, graded loading, neuromuscular retraining, and behavioral support) that aim to optimize tissue capacity and function. We define periodized rehabilitation as the planned sequencing of rehabilitation mesocycles/phases with explicit goals, dosing parameters, objective endpoints, and criterion-based progression rather than fixed timelines.

Because post-orthobiologic rehabilitation protocols remain heterogeneous and high-quality direct trials are limited, this framework is evidence-informed and clinically reasoned. When constructing phase goals and progression criteria, we prioritized (1) human clinical trials and systematic reviews of orthobiologics and rehabilitation when available; (2) human mechanobiology/mechanotherapy evidence relevant to musculoskeletal tissues; (3) clinical guidelines/consensus statements where higher-level evidence is absent; and (4) animal studies to support biological plausibility only (not to prescribe human timelines or loading thresholds).

## 2. Periodization in Rehabilitation Means a Plan for Rehabilitation

The American Physical Therapy Association defines regenerative rehabilitation as the integration of interventional orthobiologic techniques coupled with appropriate rehabilitation protocols that harness the body’s innate healing mechanisms through movement to augment the orthobiologic intervention [6,7]. Periodization may also be beneficial by adding variation to workouts by manipulating sets, repetitions, exercise order, number of exercises, resistance, rest periods, contraction type, or training frequency. The “classic” or “linear” periodization (LP) model is based on changing exercise volume and loading across several predictable mesocycles [8,9]. The program is broken down into distinct blocks named according to time frames, microcycle, or mesocycle. The LP model is based on changing exercise volume loading across several predictable mesocycles. The other main model is the non-linear or “undulating” periodization model [10,11].

Non-linear periodization (NP) is based on the concept that volume and load are altered more frequently (daily, weekly, biweekly) to allow the neuromuscular system to experience more extended recovery periods. In contrast, lighter loads are performed more often. In the NP model, stimuli change more frequently. Most rehabilitation protocols follow this approach.

## 3. Soft Tissues Need Peace and Love

Rehabilitation of soft-tissue injuries can be highly complex. Over the years, the management of acronyms has evolved from ICE to RICE, then to PRICE, and finally to POLICE [6]. These widely known acronyms focus on acute management, ignoring the subacute and chronic stages of tissue healing and predisposing to failed outcomes. These methods focus on the acute management of soft tissue injuries and do not really provide any information on the sub-acute and chronic stages of soft tissue healing [12].

More new acronyms have been introduced to optimize soft tissue recovery: PEACE and LOVE. These two acronyms (PEACE and LOVE) encompass the full range of soft-tissue injury management, from immediate care to subsequent management.

To connect acute management to orthobiologic biology, we emphasize that the PEACE components of Protection and Education can be used to safeguard the first week after PRP (a period often described as biologically active for growth-factor signaling), by avoiding unnecessary high-shear loading and by coaching patients on expected symptom behavior and appropriate activity pacing.

A proper dose of Exercise helps to restore mobility, strength, and proprioception early after injury. Painful exercises should be avoided to ensure optimal repair during the subacute phase of recovery and should be used as a guide for [6] rehabilitation progressions [6].

Managing soft-tissue injuries is more than short-term damage control. As with other injuries, clinicians should aim for long-term outcomes and treat the patient rather than the injury itself.

### 3.1. Pain, Threat, and the Nervous System “Gate”

After an injection, many people expect pain to vanish once the tissue “heals.” However, pain is an output of the nervous system, not a direct measurement of tissue damage. Nociceptors in the joint, tendon, or fascia send signals upward, but the brain decides how much danger those signals represent. If the brain predicts that movement is unsafe—based on prior flare-ups, fear, poor sleep, stress, or deconditioning—it may amplify pain and stiffness as a protective strategy. This is why two people with similar imaging can feel totally different levels of pain [13].

Orthobiologics may reduce inflammatory mediators and calm peripheral nociceptor sensitivity, but rehabilitation will teach the nervous system that graded movement is safe again. Early-stage rehabilitation often succeeds when it lowers threat: clear education for the patient in modern pain neuroscience, predictable tissue loading, and achievable exercises that end before symptoms spike. The nervous system learns through patterns—if every session ends with a flare, the brain updates its prediction to “movement equals danger,” and it will tighten the brakes next time (more pain, more muscle guarding) [14].

### 3.2. Muscle Guarding and Altered Motor Control

Pain changes movement almost immediately. The nervous system reduces force output and alters coordination to protect the region. This is not “weakness” in the classic sense; it is often inhibited. For example, knee pain and edema can downregulate quadriceps activation (arthrogenic muscle inhibition, AMI), while hip pain can reorganize gluteal activation patterns [13,14,15]. The protective strategy is helpful in the short term. However, if it persists, it creates overload elsewhere: the ankle stiffens, the low back takes more work, the other limb compensates, and the original tissue never experiences the graded mechanical stimulus it needs for full recovery [15].

Rehabilitation after orthobiologics, therefore, has a nervous system goal: restore efficient motor control and normalize protective co-contraction. Early interventions may look deceptively simple—submaximal isometrics, controlled-range work, tempo exercises, and breathing strategies—but they work because they reintroduce output with a low perceived threat. Once the nervous system allows cleaner activation, the biologically “improved” tissue can receive progressive mechanical loading—the key driver of remodeling [16].

### 3.3. Sensory Recalibration: Proprioception, Balance, and Body Maps

Orthobiologic injections are typically delivered to structures rich in sensory receptors, such as tendons, ligaments, and joint capsules. These tissues continuously feed the nervous system for important information about position, tension, speed, and function. Pain and swelling distort this input. The brain’s internal “map” of the body becomes noisier, and fine motor control suffers. That is why someone can feel “unstable” even without structural instability.

A crucial part of post-orthobiologics rehabilitation is sensory retraining: improving the quality of afferent input and the brain’s interpretation of it. Balance and proprioception drills, noise and vision perturbations, closed-chain control and open-chain exercises, and eyes-open/eyes-closed tasks are not just “conditioning.” They are nervous system calibration until complete recovery. When sensory accuracy improves, the brain reduces protective tone, allowing faster, more confident movement and dual-tasking drills [17].

### 3.4. Load Tolerance Is a Brain-And-Tissue Negotiation

Tissue adapts to load, but the nervous system decides whether you are allowed to load. After orthobiologics, you want enough mechanical stimulus to drive remodeling, but not so much that you trigger a strong protective response. This creates a negotiation between biology and neurology.

A helpful way to think about it is a “traffic light” model governed by symptoms and recovery (Table 1):

- Green: mild discomfort during rehabilitation that settles quickly and does not worsen the next day → nervous system accepts the input; progress is safe.
- Yellow: pain sensation that lingers, stiffness the next morning, or reduced confidence → the system(brain) feels uncertain; adjust the micro cycles with changes in volume, range, speed, or complexity.
- Red: sharp pain, severe morning stiffness, swelling, night pain, or significant next-day regression → threat response is high; reduce load, adjust micro cycles, and rebuild safety.

This is not just symptom management—it is motor learning—the nervous system updates based on what happens after the session. If the person recovers well, the brain reaction shifts toward safety, and you can progress faster.

The function of the autonomic nervous system is crucial for recovery, inflammation, and readiness. Rehabilitation is not only about voluntary movement, exercise dosing, or fancy tasks and exercises. The autonomic nervous system regulates not only sleep but also stress hormones, heart rate variability, immune activity, and even pain sensitivity and threshold. A highly sympathetic “fight-or-flight” tone can increase pain, impede tissue recovery, and impair motor learning. After orthobiologics, this matters because the biologic intervention aims to shape the tissue environment; chronic stress, poor sleep, and fascia in everyday movements (gait) can push that environment in the wrong direction, delaying recovery and causing disappointment for the patient [18].

Effective rehabilitation programs, therefore, manage outcomes by focusing on sleep quality, pacing, nutrition, and recovery strategies that support parasympathetic balance and function. Clinically, when a patient sleeps better and feels more relaxed, their pain threshold often improves, and their movement becomes smoother—because the nervous system is more willing to permit loading and avoid the threat of signaling.

Motor learning: rebuilding skill and proper movement, not just strength

Rehabilitation after orthobiologics should be framed as skill acquisition. Strength matters, but the nervous system must re-learn how to use parameters like strength, sequencing, and context.

Motor learning principles apply:

- Start with constraints that simplify the task (stable positions, slow tempo).
- Use high-quality, low-volume repetitions rather than high fatigue early on.
- Progress movement complexity: range → speed → load → reactivity → sport-specific chaos.
- Vary practice once basics are stable, so the nervous system generalizes to real life or returns to sport.

This is why “perfect form” at slow speed does not automatically translate to sprinting, cutting, or lifting under fatigue. The nervous system needs progressive exposure to the exact constraints of the goal of the activity.

### 3.5. The Role of Expectations and Placebo/Nocebo

Orthobiologics sit in a space where expectations can be mighty. Positive expectations can reduce perceived threat and improve engagement; negative expectations (“this will not work,” “my tendon is damaged”) can amplify protective nervous system responses. This is not about “imagining” recovery—it action.

The brain constantly predicts outcomes and protects against threats (inaccurate periodization scheme?); rehab succeeds when predictions align with graded, successful experiences [19].

Evidence-based clinicians leverage this by setting realistic timelines, explaining flare-ups, and emphasizing controllable progress markers (range, capacity, confidence, function). That messaging directly changes how the nervous system interprets training inputs.

A practical guide for recovery after orthobiologics often follows three overlapping phases:

For structural consistency, we refer to these as three overlapping macro-phases (Calm, Reload, Re-skill) that map onto the five-phase periodized model (Phases 1–5) detailed below. The macro-phases communicate the clinical intent, whereas Phases 1–5 operationalize dosage, objectives, and criterion-based progression (Table 2).

- Calmand re-engage (days to weeks depending on tissue/site, age, and medical history): low-threat movement, isometrics, gentle range, education, symptom monitoring. Goal: reduce guarding and restore basic movements (etc. gait, stairs, climbing)
- Reload and re-coordinate (weeks): progressive strength with motor control, proprioception, and graded exposure to movements that were threatening before the treatment. Goal: Normalize motor patterns and increase load tolerance.
- Re-skill and return to sport (weeks to months): speed, plyometrics, perturbations, sport/work specificity, fatigue resistance, decision-making, dry land: nervous system confidence under real-world conditions.

Across all mesocycles, the nervous system is the “operating system” that determines how the body can express tissue movements and healing that orthobiologic treatments may support. When rehabilitation and sports medicine respect modern pain neuroscience, rebuild sensory re-writing, and use motor learning rules, the injection of an agent becomes a catalyst rather than a standalone fix. The real endpoint is not just less pain—it is a nervous system that trusts the tissue remodeling again, allowing strength, speed, and skill to return to sport safely and sustainably [20].

## 4. Rehabilitation Mesocycles/Phases Following Regenerative Medicine Procedures in Orthopedic Patients

Research on rehabilitation protocols for regenerative procedures in orthopedic patients is scarce, with no standard protocols for rehabilitation after interventional orthobiologic procedures in humans [16]. However, animal studies corroborate the Mechan transduction model for promoting healing. In the equine population undergoing PRP injections, controlled gradual return to activity or sport is the best course of action, with restricted Exercise in the acute and subacute phases of tendon and ligament healing being paramount [17].

Translational note: Animal and in vitro studies are used here to illustrate Mechan transduction principles (i.e., that cells respond to mechanical stimuli), but differences in species, injury models, loading environments, and outcome measures limit direct translation to clinical dosing. Therefore, human rehabilitation decisions in this framework are criterion-based and anchored to symptom irritability, functional measures, and validated clinical assessments rather than extrapolated animal timelines.

Rehabilitation appointments begin 10–14 days after the procedure, to protect the affected tendon or joint and control pain. Patients must follow clear instructions after the orthobiologics procedure. First, rest and healing are important after PRP injections. The injected PRP should be allowed to heal the affected area. For this to happen, the PRP must be allowed to release growth factors and proteins that promote tissue regeneration and healing [18]. It takes up to 7 days for growth factors to be released from platelets [19]. Exercise may displace and move the PRP from the injected site, compromising healing and outcome. Rest for the first two weeks, followed by a gradual return to regular Exercise, is ideal, with close monitoring of pain and swelling.

Ice as a recovery tool should not be used after PRP injections. Inflammation is an important stage of the healing process. Ice reduces inflammation and swelling and may therefore compromise healing [20]. Ice also constricts blood vessels, thereby reducing blood flow.

Also, the patient must be instructed not to use non-steroidal anti-inflammatory drugs.

As with many injuries treated with orthobiologics, the recovery protocol will vary, and the time to recovery will differ. However, for a PRP injection into the joint, recovery time is usually 8–12 weeks. This means that joint injuries treated with platelet-rich plasma will take approximately 3 months before patients can run freely again, with some interindividual variability.

Trying to speed up recovery time after injection into the joint can often cause the recovery time to increase.

A rehabilitation protocol with a periodization structure can be like the following one:

Phase 1 (Mesocycle) should begin immediately after the procedure and can last up to 1 week, depending on pain and inflammation. In the acute mesocycle, it is advantageous to begin weight-bearing and loading as soon as possible and tolerable. Early gentle mobility exercises and tissue loading will benefit tissue remodeling and healing. General rehabilitation guidelines for orthobiologics involve four phases of therapy [21]. The following example applies the four-phase approach to the rehabilitation of a patient with various joint pathologies after PRP injection, which can be extrapolated to stem cell therapies, though typically with a longer course of action to allow for healing.

The rehabilitation program should incorporate strength, stability, and intermuscular balance of the core, hips, and surrounding joints, using a holistic approach. Early implementation of these corrective exercises will prepare the body for the progression of joint-loading exercises and more complex tasks. Exercises will progress to functional multi-joint exercises, focusing. Phase 1 is critical for future recovery and will focus on reducing pain and inflammation through early-loading exercises and mechanotherapy, as tolerated. Complete functional weight-bearing Exercise should include squats, lunges, single-leg step-downs, glute bridges, and single-leg deadlifts. Exercise periodized progressions will depend on the patient’s neuromuscular control, age, medical history, functional mobility, and exercise tolerance (Table 3).

Phase 2 (Mesocycle) can begin once pain and inflammation are much reduced, and neuromuscular control and static stability are at adequate levels.

Phase 3 (Mesocycle) can begin once the individual has achieved full mobility.

Phase 4 (Mesocycle) can be initiated when the individual attains a non-painful range of motion. The primary treatment modalities include active rehabilitation to promote tissue healing, in conjunction with PRP or stem cell therapy—early mobilization and, in the acute phase, loading as pain permits. A graded load of progression and cardiovascular Exercise are recommended for all patients.

Phase 5 (Mesocycle) will be focused on neuroplastic rehabilitation, including exercises to stimulate neurogenesis, angiogenesis, and synaptogenesis, that enhance neural plasticity and ameliorate some of the deleterious morphological and behavioral sequelae of aging. The hippocampus is a primary target of exercise effects in the brain [22], with Exercise shown to alter hippocampal structure and/or function across both rapid and longer-term time spans and age groups [23,24]. Brain-derived neurotrophic factor (BDNF) is a neurotrophin that supports neurogenesis, promotes neuronal survival and synaptic plasticity, and is highly expressed in hippocampal neurons. The expression of specific BDNF levels can be regulated by epigenetic mechanisms, suggesting that environmental experiences can dynamically influence mature BDNF levels. In both young and aged rodents, Exercise increased BDNF expression in the hippocampus and cortical regions, with effects persisting for several weeks after exercise [25].

**Figure 1:**
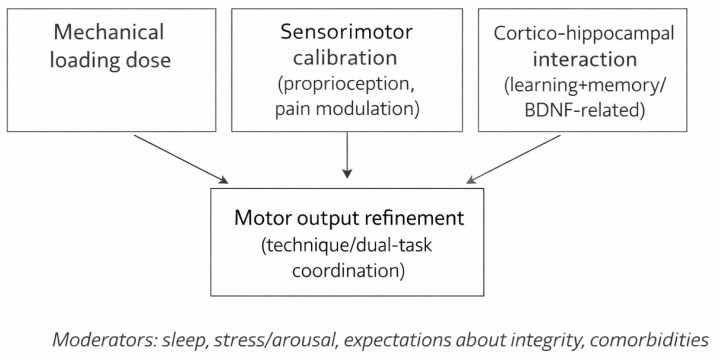
Neuroplastic periodization loop linking mechanical loading to sensory recalibration and learning/memory processes (conceptual).

Descriptions of the five phases of rehabilitation, in general, after an orthobiologic procedure application to orthopedic patients are the following (Figure 1):

### 4.1. Phase 1

It begins during post-injection day 0 to 3, and the goal is protection of the injected region and pain control. Immobilization and/or complete unloading (non-weight-bearing status) of the affected joint should be avoided in the lower extremity. A sling may be considered for shoulder pain, and an unloading brace for knee osteoarthritis (OA).

Partial weight-bearing with crutches for hip pathologies and the use of a walking boot for ankle/foot pathologies may be utilized. A gentle range of motion out of the immobilizing device should be performed passively and actively (to tolerance) for short durations (2–3 min), multiple times per day (3 times).

### 4.2. Phase 2

After days 4 to 14, the goal should be to increase tissue tolerance to loading, discontinue immobilization/unloading devices, and slowly progress to weight bearing while avoiding shear stress. Continue active and passive range of motion activities for 3–5 min/session, 3–5 times a day.

Begin submaximal isometric exercises for affected tendons/joints, begin progressive loading for lower extremity pathologies, unloaded cycling, and core stability exercises.

Proceed to the next level if the pain score is 2 or lower on a visual analog scale.

In addition to pain ratings, clinicians should consider swelling/effusion, ROM targets, strength capacity (e.g., handheld dynamometry or isokinetic testing when available), movement quality on functional tasks, and next-day response (return to baseline within 24 h). Early loading should be graded and low in shearer; potential risks of premature or excessive loading include symptom flare, reactive synovitis/effusion, tendon overload, and compensatory movement strategies that may delay recovery and reduce adherence.

### 4.3. Phase 3

Encompasses weeks 3–6 to restore the full range of motion to the affected joint, increase tissue tolerance to loading, and improve strength/endurance. For knee and hip OA, the patient can walk as tolerated, start intermittent jogging, and experience no more than mild pain (2/10).

For shoulder, knee, and hip OA, patients can start with upper-body light exercises, along with modified yoga and biking.

### 4.4. Phase 4

Encompasses weeks 7+ to return to complete activities that were being performed prior to the procedure. No strict restrictions are advised; a gradual return to full functional activity is recommended. The target for 100% of desired activities is 8–12 weeks.

### 4.5. Phase 5

Begin eccentric exercises with dual tasking (starting eccentric exercises too soon can halt the healing cascade by preventing angiogenesis due to excessive load).

High-load eccentric training is introduced in Phase 5 (≈Months 3+) for most indications. However, submaximal, tempo-controlled eccentric bias (e.g., low-load eccentrics within pain limits, longer time-under-tension, assisted eccentrics) may be initiated in late Phase 3–4 only if irritability criteria are met (no effusion escalation, next-day return to baseline within 24 h, and adequate movement quality). The intent is to avoid early excessive loads that could aggravate symptoms or disrupt vascular remodeling.

It is recommended to keep the pain scale to 2–3/10 and start with two repetitions, gradually increasing to 10.

Progress to weight-bearing functional activities in the lower quarter. Begin functional retraining of the upper quarter in patient-relevant methods (weight bearing, plyometrics, etc.)

The viscoelastic properties of muscle are continuously adjusted depending on the anticipated functional demands (e.g., landing, cutting, decelerating) [26]. The neural origin of this ‘fine muscle tuning’ exerts a net effect on muscle contractions that can increase joint stiffness tenfold, maximizing performance while preserving joint equilibrium and stability [27,28]. To reduce stiffness during each task, the surrounding physical environment must be quickly modeled in the brain before executing athletic maneuvers. This process is largely unconscious, and, in fact, conscious “overthinking” and inordinately high arousal levels may delay or interrupt routine functional tasks [29,30].

At months 3+, reassess improvement and if not higher than 75% improved from the original injury, consider repeat injection and return to Phase 1.

## 5. Limitations and Applicability

This periodized framework is intended as a criterion-based template rather than a rigid, time-driven protocol. It is informed by available human evidence and mechanobiology principles, but direct post-orthobiologic rehabilitation trials remain limited, and procedures/indications are heterogeneous. As with any expert-opinion framework, potential sources of bias include selective evidence of emphasis and publication bias; therefore, prospective validation and condition-specific refinement are needed. For older or non-athletic populations, progression may be slower, and endpoints should prioritize activity of daily living, balance/fall-risk, comorbidity screening, and strength capacity rather than sport-specific performance metrics.

## 6. Conclusions

A rehabilitation framework that operationalizes phase goals, measurable endpoints, and progression criteria after orthobiologic interventions. The framework advances current practice by (i) explicitly linking mechanotherapy dose and motor-learning/neuroplastic rehabilitation to functional outcomes, (ii) embedding objective assessments (pain/irritability response, ROM, strength, functional testing, and validated patient-reported measures) to support transparent decision-making, and (iii) defining safeguards that mitigate risks of premature high-shear or high-volume loading. While the phase structure provides standardization, phase entry and exit are individualized based on clinical criteria rather than fixed timelines. The main strengths are clarity, reproducibility, and measurement-oriented progression; the main limitations are heterogeneity of orthobiologic procedures and the current scarcity of direct post-intervention rehabilitation trials, underscoring the need for prospective validation and condition-specific protocols.

Interventional orthobiologics still need to develop a consensus on the procedures to use for specific patients and on recommendations for physical therapy management after procedures. Such recommendations need to be patient-specific and correlated with the mechanobiology of the body segment or tissue treated. Orthobiologics treatments are focused more specifically on stimulating regenerative potential. To maximize the clinical success of these regenerative procedures, standardization of preparation, administration, and post-injection treatment protocols is imperative. The framework for the rehabilitation program described in the present article is based on current scientific evidence and clinical expertise related to rehabilitation for individuals who have various musculoskeletal disorders and who have been treated with orthobiologics.

## Figures and Tables

**Table 1:** Traffic-light clinical decision guide for graded loading after orthobiologic procedures.

Zone	Typical Clinical Presentation	Load/Action	Monitor/Progression Trigger
Green	Symptoms stable and low; no meaningful next-day flare; movement quality acceptable.	Progress loading (low-shear → higher-shear) and add complexity (speed/perturbation) as appropriate.	Objective targets met (ROM/strength/function) and return to baseline within 24 h.
Yellow	Irritable or fluctuating symptoms; mild effusion/swelling; next-day increase but resolves.	Maintain or slightly regress dose; emphasize technique, tempo, and recovery; avoid high-shear spikes.	Proceed when symptoms stabilize and functional quality improves across sessions.
Red	Escalating pain, night pain, marked swelling/effusion, or worsening function.	Stop aggravating loads; return to earlier phase inputs; consider medical reassessment/red flags.	Resume progression only after red-flag exclusion and clear symptom down-trend.

**Table 2:** Alignment of biological stage, macro-phase (Calm/Reload/Re-skill), and operational Phases 1–5.

Biological Emphasis (Simplified)	Macro-Phase	Operational Phase	Primary Rehab Focus/Examples
Post-procedure irritability; early biological signaling	Calm	Phase 1 (Days 0–3)	Protect region; symptom modulation; gentle ROM; avoid unnecessary shear
Settling inflammation; restoring tolerance to load	Calm → Reload	Phase 2 (Days 4–14)	Isometrics; low-shear loading; mobility; re-engage gait/function; education/pacing
Capacity building and tissue tolerance	Reload	Phase 3 (Weeks 3–6)	Strength/endurance; full ROM; graded exposure; aerobic base; movement quality
Integration to higher function	Reload → Re-skill	Phase 4 (Weeks 7+)	Return to prior activities; progressive complexity; controlled plyometrics/perturbations as appropriate
Neuroplastic re-skill and performance resilience	Re-skill	Phase 5 (≈Months 3+)	Dual-tasking; sensory recalibration; sport/work specificity; fatigue robustness; return-to-performance

**Table 3:** Operationalized phase structure with example endpoints, progression criteria, and safeguards (examples should be adapted to tissue, indication, and patient population).

Phase	Primary Goals	Example Interventions	Objective Endpoints (Examples)	Progression Criteria (Examples)	Key Risks/Safeguards
Phase 1 (Days 0–3)	Protection, pain/irritability control	Relative rest, protected ROM, education	NRS/VAS, effusion/swelling, ROM tolerance	Symptoms stable; no worsening in 24 h; acceptable swelling	Avoid aggressive loading; monitor night pain/effusion
Phase 2 (Days 4–14)	Restore tolerance to low load	Submap isometrics, unloaded cycling, basic control	ROM targets, HHD baseline, functional quality	Pain < 3/10 and returns to baseline < 24 h; ROM improving	Avoid reactive flare; adjust load/frequency
Phase 3 (Weeks 3–6)	Full ROM; progressive strength	Isotonic, closed-chain, gradual exposure	Strength (HHD/isokinetic), LEFS/KOOS/HOOS/Quick DASH	Strength progressing; functional tasks without compensation	Avoid compensations; monitor delayed soreness
Phase 4 (Weeks 7+)	Return to pre-procedure activity	Higher-load strength, plyometrics as indicated	Hop/jump tests (athletes), power metrics	Objective thresholds (e.g., ≥90% symmetry)	Load spikes; ensure graded exposure
Phase 5 (Months 2–3+)	Neuroplasticity + RTP/performance	Dual-task, perturbation, eccentric, agility	Y-Balance/SEBT, dual-task metrics, ACL-RSI/TSK (as relevant)	Criterion-based RTP; next-day response stable	Avoid premature high shear; fatigue monitoring
